# Bevacizumab-Induced Reversible Thrombocytopenia in a Patient with Adenocarcinoma of Colon: Rare Adverse Effect of Bevacizumab

**DOI:** 10.1155/2012/695430

**Published:** 2012-10-10

**Authors:** Jeevan Kumar, Manorama Bhargava, Shyam Aggarwal

**Affiliations:** ^1^Sir Ganga Ram Hospital, New Delhi 110060, India; ^2^Department of Hematology, Sir Ganga Ram Hospital, Old Rajinder Nagar, New Delhi 110060, India

## Abstract

We report a case of bevacizumab- (BEV-) induced thrombocytopenia in a 59-year-old man with adenocarcinoma of colon. After colectomy, the patient was treated with twelve cycles of FOLFOX-4 (folinic acid, 5-fluorouracil, and oxaliplatin) regimen. On relapse, he was treated with FOLFIRI (folinic acid, 5-fluorouracil, and irinotecan) regimen along with BEV 10 mg/kg for 6 cycles. After that, BEV was continued for maintenance as a single agent at an interval of three weeks. After the13th cycle of BEV, the patient developed melena with epistaxis and thrombocytopenia, from which he recovered on withdrawal of BEV. On rechallenge with half the initial dose, there was once again a reversible drop in platelet count. The proposed mechanism of thrombocytopenia may be immune-mediated peripheral destruction of platelets.

## 1. Introduction

Bevacizumab (BEV) is a humanized immunoglobulin monoclonal antibody that binds to and inhibits the activity of vascular endothelial growth factor (VEGF). It can result in two distinct patterns of bleeding: minor hemorrhage, most commonly grade 1 epistaxis; serious, and in some cases fatal, hemorrhagic events. Severe or fatal hemorrhage, including hemoptysis, gastrointestinal bleeding, hematemesis, CNS hemorrhage, epistaxis, and vaginal bleeding has been reported up to fivefold more frequent in patients receiving BEV compared to those receiving chemotherapy alone [1–3]. BEV also impairs wound healing. In a controlled clinical trial, the incidence of wound healing complications in patients with metastatic colorectal cancer who underwent surgery during the course of BEV treatment was 15% as compared to 4% in those who did not receive BEV. Serious and sometimes fatal gastrointestinal perforation is more frequent in BEV-treated patients compared to controls [1]. We report here the case of a 59-year-old man with relapsed adenocarcinoma of colon. After surgery and multiple chemotherapies, BEV was continued as a single agent. After the 13th cycle of BEV, the patient suffered from melena, epistaxis, and thrombocytopenia.

## 2. Case Report

In June 2008, the patient presented with subacute intestinal obstruction. On investigations, he was found to have mucin-secreting well-differentiated adenocarcinoma of colon Duke stage C. There were no liver nodules, ascites, and peritoneal dissemination. After hemicolectomy, the patient was treated with twelve cycles of FOLFOX-4 (folinic acid, 5-fluorouracil, and oxaliplatin) regimen. He received the last cycle of chemotherapy in February 2009. A CT scan done after the completion of chemotherapy showed no mass or enlarged lymph nodes. Postchemotherapy carcinoembryonic antigen (CEA) was 8 *μ*g/L (0–5 *μ*g/L).

The patient remained asymptomatic till February 2010, when he developed abdominal pain and ascites. PET-CT showed omental thickening with few small nodular deposits in anterior abdominal wall with moderate ascites. Biopsy of nodular deposits in anterior abdominal wall and ascitic fluid examination revealed adenocarcinoma. The blood investigations were CEA = 74.8 *μ*g/L, Hb = 10.9 g/dL, TLC = 9,000/μL, and platelet count = 212,000/*μ*L. This relapse was treated with FOLFIRI (folinic acid, 5-fluorouracil, and irinotecan) regimen along with BEV 10 mg/kg for 6 cycles, last cycle in July 2010. The patient responded quite well to this regimen. The PET-CT scan showed reduction in omental thickening and ascites. Investigations after chemotherapy were CEA = 11 *μ*g/L, Hb = 12.8 g/dL, TLC = 7,880/μL, and platelet count = 191,000/*μ*L. After that, BEV 10 mg/kg was continued as a single agent at an interval of three weeks. The patient had recurrent episodes of epistaxis after starting BEV.

In November 2010, the patient again reported with abdominal pain. CT scan of the abdomen showed extensive peritoneal and omental deposits. External beam radiotherapy was given to omental plaques. In March 2011, the patient developed jaundice. CT scan of abdomen showed dilatation of intrahepatic biliary radicals with soft tissue density lesion at the confluence of right and left hepatic ducts causing extrinsic compression. The blood investigations were Hb = 10.7 g/dL, TLC = 10,700/μL, and platelet count = 224,000/*μ*L. Patient recovered after percutaneous transhepatic biliary drainage (PTBD).

In July 2011, the patient started to have melena along with epistaxis two days after completion of the 13th cycle of BEV as a single agent. On investigations Hb was 5.2 g/dL, TLC was 14,000/*μ*L (neutrophils 85%, lymphocytes 11%, monocytes 02%, myelocytes 01%, and metamyelocytes 01%), and platelet count was 10,000/*μ*L. A repeat platelet count on the same day was 6,000/*μ*L. Bone marrow examination showed cellular marrow with all hemopoietic elements with increased megakaryocytes. No bone marrow infiltration was seen. The patient was receiving no other known medications that could be linked to the development of drug-induced thrombocytopenia. He did not have a central venous access and was not receiving heparin. Antiplatelet antibodies were absent on two separate occasions. Other hematologic parameters, fibrinogen, fibrin monomer, D-dimer, PT/aPTT, peripheral smear, bilirubin, LDH, antinuclear antibodies (ANAs), and haptoglobin were unremarkable on two separate occasions.

**Figure 1:**
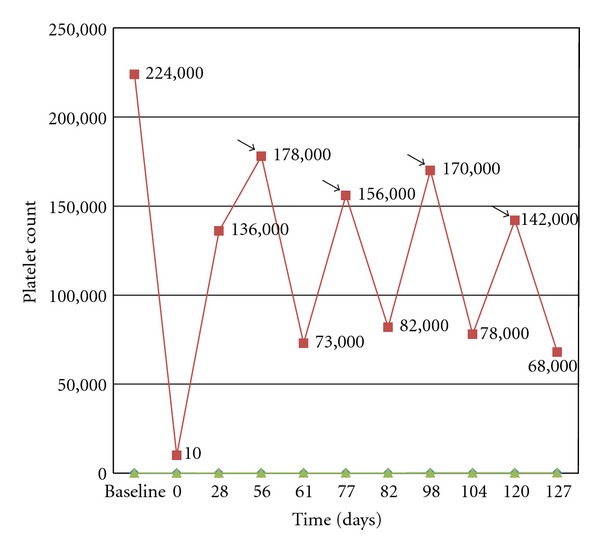
Changes in platelet count in relation to treatment with Bevacizumab. Arrows indicate injection of bevacizumab (5 mg/kg).

For epistaxis nasal packing was done. One unit platelet apheresis and two units of packed red blood cells were transfused. Two additional units of packed red blood cells were transfused the next day. BEV was discontinued. The patient was given injection methylprednisolone 1 g intravenously daily for three days. Epistaxis and melena stopped on the second day. Platelet count on the second day was 38,000/*μ*L. His platelet count started rising, and after 4 weeks it was 136,000/*μ*L. After 8 weeks, when his platelet count was 178,000/*μ*L, he was rechallenged with half the dose (5 mg/kg) of BEV (Figure 1). There was once again a drop in platelet count to 73,000/*μ*L, which rose to 156,000/*μ*L after 3 weeks. Again 5 mg/kg of BEV was given, and platelet count dropped to 82,000/*μ*L. Since the platelet count recovered after 3 weeks, BEV 5 mg/kg was continued every 3 weeks (if platelet count was above 100,000/*μ*L) along with monitoring of platelet count.

## 3. Discussion

Our patient developed melena with epistaxis and thrombocytopenia, attributable to BEV. Thrombocytopenia is a rare adverse effect of BEV that has only few case reports in the literature. Leal et al. reported a case of bevacizumab-induced reversible thrombocytopenia in a patient with recurrent high-grade glioma [4]. In their case report, the patient had no bleeding and platelet count was mildly dropped, but our patient had melena, epistaxis with severe thrombocytopenia.

Bevacizumab is a recombinant humanized monoclonal neutralizing antibody against vascular endothelial growth factor (VEGF), which has shown clinical benefits and efficacy in several types of malignancies including metastatic colorectal and lung cancer [2]. Treatment with BEV is associated with increased rates of arterial and venous thromboembolism and hemorrhage. In a large observational treatment study in patients with metastatic colorectal cancer, the incidence rate of clinically significant bleeding associated with BEV was 2.4% [5]. 

In the study of Hurwitz et al. [2, 3], grade 1 or 2 hemorrhagic events were more frequent in patients receiving irinotecan, bolus fluorouracil, and leucovorin (bolus-IFL) plus BEV when compared to those receiving bolus-IFL plus placebo and included gastrointestinal hemorrhage (24% versus 6%), minor gum bleeding (2% versus 0), and vaginal hemorrhage (4% versus 2%). Incidence of epistaxis was higher (35% versus 10%) in patients receiving bolus-IFL plus BEV compared with patients receiving bolus-IFL plus placebo. Impaired wound healing had contributed to these hemorrhagic events. Incidence of grade 1 or 2 thrombocytopenia was higher (5% versus 0%) in patients with metastatic colorectal cancer receiving bolus-IFL plus BEV compared with patients receiving bolus-IFL plus placebo [2, 3]. Various clinical trials showed that addition of BEV to chemotherapy did not significantly alter the incidence of thrombocytopenia [6–8].

The pathophysiological mechanisms leading to these side effects are poorly understood. Data from in vitro experiments and animal models point to a possible influence of bevacizumab in primary hemostasis and platelet function. Recently VEGF and VEGF receptors (VEGF-Rs) have been found to be relevant mediators of platelet aggregation [9, 10]. Both of these targets represent potential sites at which bevacizumab could potentially interact with primary hemostasis. 

A proposed mechanism of thrombocytopenia was described by Meyer et al. [10]. BEV forms immune complexes (ICs) with VEGF, a heparin-binding protein. In presence of heparin, BEV+VEGF immune complexes activate platelets via the IgG receptor Fc*γ*RIIa—a mechanism similar to that observed with antibodies from patients with heparin-induced thrombocytopenia (HIT). VEGF can directly anchor to platelet surface-bound platelet factor-4 (VEGF binds platelet factor-4 with high affinity) [11], which may explain heparin-independent BEV+VEGF activity. Meyer et al. [10] provided evidence that BEV immune complexes can directly induce platelet aggregation and granule release in vitro and cause thrombocytopenia and thrombosis in vivo in a murine model.

Thrombocytopenia can also be because of bevacizumab causing platelet dysfunction and consumption leading to shortened platelet half-life. It seems that overt thrombocytopenia would then develop once the compensatory mechanisms of the bone marrow became exhausted, particularly in a patient who has had multiple prior therapies.

A meta-analysis by Kut et al. [12] concluded that VEGF in cancer patients is mostly concentrated in the platelets within the blood compartment and that the cancer itself is not the main source of VEGF in the body. In vitro tests have shown a stimulatory effect of VEGF on thrombin-induced platelet activation. This suggests that the endogenously secreted platelet VEGF may function as a positive feedback regulator during platelet activation [9]. Theoretically, the interaction of bevacizumab with the platelet VEGF during platelet activation could result in impaired primary hemostasis, increasing the risk of hemorrhage.

Since the platelet counts recovered after methylprednisolone and bone marrow had shown increase in megakaryocytes, the proposed mechanism of thrombocytopenia in our patient may have been immune-mediated peripheral destruction of platelets. It is of course possible that other mechanisms could also have contributed to thrombocytopenia as an additive effect.

High degree of awareness is required to this potential complication, which may have significant implications for clinical care and ongoing research.
